# A Simpler Energy Transfer Efficiency Model to Predict Relative Biological Effect for Protons and Heavier Ions

**DOI:** 10.3389/fonc.2015.00184

**Published:** 2015-08-11

**Authors:** Bleddyn Jones

**Affiliations:** ^1^Gray Laboratory, CRUK/MRC Oxford Insitute for Radiation Oncology, University of Oxford, Oxford, UK

**Keywords:** RBE, protons, ions, radiotherapy, radiobiology

## Abstract

The aim of this work is to predict relative biological effectiveness (RBE) for protons and clinically relevant heavier ions, by using a simplified semi-empirical process based on rational expectations and published experimental results using different ion species. The model input parameters are: *Z* (effective nuclear charge) and radiosensitivity parameters α_L_ and β_L_ of the control low linear energy transfer (LET) radiation. Sequential saturation processes are assumed for: (a) the position of the turnover point (LET_U_) for the LET–RBE relationship with *Z*, and (b) the ultimate value of α at this point (α_U_) being non-linearly related to α_L_. Using the same procedure for β, on the logical assumption that the changes in β with LET, although smaller than α, are symmetrical with those of α, since there is symmetry of the fall off of LET–RBE curves with increasing dose, which suggests that LET_U_ must be identical for α and β. Then, using iso-effective linear quadratic model equations, the estimated RBE is scaled between α_U_ and α_L_ and between β_U_ and β_L_ from for any input value of *Z*, α_L_, β_L_, and dose. The model described is fitted to the data of Barendsen (alpha particles), Weyrather et al. (carbon ions), and Todd for nine different ions (deuterons to Argon), which include variations in cell surviving fraction and dose. In principle, this new system can be used to complement the more complex methods to predict RBE with LET such as the local effect and MKM models which already have been incorporated into treatment planning systems in various countries. It would be useful to have a secondary check to such systems, especially to alert clinicians of potential risks by relatively easy estimation of relevant RBEs. In clinical practice, LET values smaller than LET_U_ are mostly encountered, but the model extends to higher values beyond LET_U_ for other purposes such as radiation, protection, and astrobiology. Considerable further research is required, perhaps in a dedicated international laboratory, using a basket of different models to determine what the best system or combination of systems will be to make proton and ion beam radiotherapy as safe as possible and to produce the best possible clinical results.

## Introduction

Positively charged particle therapy is increasing worldwide. Its numerous potential advantages in cancer therapy depend on the Bragg peak effect (1–4), but the increase in linear energy transfer (LET) causes enhanced biological effects which change normal tissue tolerances, as well as tumor control probabilities. LET, typically reported as kiloelectron volt per micrometer, refers to the ratio of energy released from a radiation beam per unit micrometer track length and is used as a measure of radiation quality. It can be expressed in two different ways, either as the mean or the dose averaged LET. Relative biological effect (RBE) is defined as the ratio of dose of a low LET radiation divided by the control high LET dose required for the same biological effect. RBE, although measured quite simply in this way, depends on the complexities of how radiation of different qualities interact with different biological systems due to:

(1) The energy, depth, and mixture of Bragg peak or non-Bragg peak regions over a volume of interest.
(2) The increased local complexity, or clustering, of DNA damage with increasing LET (5).
(3) The increasing difficulty in repairing the more clustered damage, resulting in increased radiosensitivity and reduced fractionation sensitivity.

Some authors have developed relatively simple LET–RBE models for protons (6–8). For ion beams, there are several complex formulations that tentatively describe the relationship between LET and RBE (9–14), each with varying degrees of success, and have been used for clinical applications. These ion beam models are based on the fundamental interactions of particle physics with matter and contain multiple assumptions and input requirements, such as knowledge of particle trajectories relative to cells, cross sectional probabilities, the relative proportion of cell nucleus to cell volume for each cell, critical biological sub-volumes, repair capacities, and extrapolations with dose, etc. They all utilize long mathematical constructs which can be daunting to less mathematically gifted individuals. Whereas it is satisfying to build exploratory theoretical models in such a way, it is impossible to know these exact conditions within a real cancer and surrounding normal tissues. These various approaches have been used to predict ion beam RBE values for variable LET values for the irradiation of specific cell types (usually the V-79 cell derived from Chinese Hamsters), but with mixed results, although they are used routinely in clinical practice for carbon ion treatment planning. Only some authors have attempted an approach for normalizing the RBE differences between different ions, as in the work of Katz (9), who used the parameter *Z*^2^/β^2^ to calculate the radial distribution of dose (where *Z* is nuclear charge and β is the relativistic velocity).

What do we know with certainty about LET and RBE? Measured relationships between LET and RBE generally show increases with LET until a maximum value is achieved, followed by a decrease to RBE values just above unity. Also, there are important basic findings, shown by multiple authors (15–18), which are essential to incorporate into any model that adequately describes the change of RBE changing with LET. They are:

- The initial slope of RBE with LET is linear when plotted on linear scales (19).
- The LET value (LET_U_) which confers the maximum cell killing efficiency (at the turnover point) increases non-linearly with the nuclear charge of a particle (the *Z* number), which denotes the electrostatic positive charge of the particle nucleus. LET_U_ values increase with *Z*, but smaller increments in LET_U_ occur with increasing *Z*, which suggests a saturation effect. Ions with the smallest *Z* values are consequently more efficient in increasing RBE per unit increase in LET, possibly because the energy released is more locally absorbed than is the case for higher *Z* ions with larger event sizes and more energetic gamma emissions.
- The magnitude of the RBE is not only dependent on the particle type (or *Z*), but also depends on LET, dose (and so the surviving fraction of cells), and the cell type (and its ability to repair radiation damage).
- The magnitude of the RBE depends on cell type (seemingly regardless of the ion used), with cells that are intrinsically more radiosensitive (to very low LET radiations) having lower RBEs than their more radio-resistant counterparts.
- The RBE increases when the cell-surviving fraction is reduced (for lower doses), but the LET–RBE turnover point position remains constant. Thus, the overall symmetry is preserved.
- In terms of the linear quadratic model (LQ) of radiation effect, the α value increases with LET to a far greater extent than β (20–22). The relative increase in α with LET is greatest for cells/tissues which have the lowest, most radio-resistant, low LET α_L_ values, with smaller increases in α for systems which have the most radiosensitive, highest intrinsic α_L_ values, as will be shown later.
- These experimental findings apply to well-oxygenated cells, but are modified in radiologically hypoxic conditions (16), probably since the α parameter-related cell kill is not so influenced by oxygen as is the β parameter-related cell kill.

This article considers how a much “simpler LET efficiency model” can estimate the LET–RBE relationships described above and their modification with dose, *Z*, and the two low LET intrinsic radiosensitivities α_L_ and β_L_. These models require fewer input parameters and assumptions than do the far more complex models already referred to above. Such a model could be used to complement the other systems: in this sense, two predictions may carry more reliability if they are in close agreement.

## Materials and Methods

### The experimental data

There are relatively few published experiments that provide a reasonable estimate of LET turnover positions (LET_U_) for clinically used particles. These include the data sets of Belli et al. (18), Barendsen et al. (15), Furusawa et al. (16), and Weyrather et al. (17). These experiments were not designed to accurately determine LET_U_, but to show overall phenomena and to determine the range of RBE values. Inevitably, overall accuracy is further undermined by biological variation, use of different cellular assays in various laboratories, use of different LET interpretations and measurements over wide ranges with consequent use of a logarithmic scaled abscissa, which masks the uniform initial linear slope of the relationship. To obtain the best available estimate of LET_U_, only the most unequivocal examples of maximum radiosensitivities, or RBE, over a small range of LET near to LET_U_, were used. Data where LET_U_ could not be determined to reasonable accuracy, as in some of the HRG cellular data of Furusawa et al. (16) and in some carbon ion experiments, were excluded. The LET_U_ values (keV/μm) so obtained were 30.5 (protons), 103.4 (helium), 208 (carbon), and 233 (neon). Although data exist for heavier ions such as silicon and argon, these do not provide a sufficiently accurate estimate (23).

For model fitting, the experimental studies of Todd (24), using a wide range of ions (deuterons, helium, lithium, boron, carbon, oxygen, nitrogen, neon, and argon), of Barendsen (deuterons and helium) (15), and of Weyrather et al. (carbon) (17) were used to test data against the modeled predictions.

The highest α radiosensitivity obtained (α_U_), in the region of LET_U_, for each ion species, was plotted against the low LET (control) α_L_ value from the same data. These values are shown in later graphical plots, and include variation due to the LET_U_ position uncertainty. The accuracy of the β radiosensitivity parameter is less easy to determine for high LET radiations (compared with low LET radiations), for reasons discussed elsewhere (8, 22). It is known that β increases to a lesser extent than α with LET. In order to maintain the observed constant position of LET_U_ with increasing dose (and reduced surviving fraction), and the overall symmetry of the LET–RBE relationship, both α and β must follow similar functions which rise to a maximum at LET_U_. Otherwise, the overall symmetry of the LET–RBE curves with increasing dose would be broken: for example, if LET_U_ would be different for α and β, the LET_U_ would be observed to change with dose, which is not the case.

### Detailed description of model

The turnover of RBE with LET, is a well-reported phenomenon often attributed to “overkill” or wasted local dose. This process can be interpreted as increasing efficiency of cell kill in the upward phase, followed by later inefficiency. In physics terms, the number of particle trajectories crossing a cell reduces by a reciprocal function of increasing LET after the turnover point. This is necessary in order to maintain the same overall dose to a wider volume with increasing LET. At the same time, increasing LET produces greater clustering of dose deposition, but over-clustering will not necessarily lead to enhanced biological effects. In bio-physical terms, increasing LET must, initially, enhance the intrinsic radiosensitivity parameters, the increment in α far exceeding that in β (25). This is because a greater proportion of more clustered damage is non-repairable by the non-homologous end joining process, although the repair of sub-lethal damage (within the more sparsely clustered damage regions) continues, although probably with lower fidelity, and even the recombination repair mechanism may also be overwhelmed by increasingly complex lesions affecting the same sites on sister chromatids.

Ionizing radiation damage in biological systems causes a hierarchy of effects: the most commonly occurring DNA base change and single strand breaks are followed by less frequent double strand breaks, nearly all of which are repaired in the case of low LET radiation. An excess of a mixture of these forms of damage in a locality of a chromosome can lead to a chromosome break, certain types of which inevitably confers lethality. Thus, the essential lesion is the “lethal” form of chromosome break (LCB) for most forms of radiation cell death at clinical doses. The local deposition of energy that results in the maximum probability of a single LCB must represent the maximum efficiency of the system, since further energy deposition and greater DNA and chromosomal structural change in the same locality will result in no extra effect; in fact any dose deposited in excess of that required to achieve a LCB will be “wasted dose,” representing inefficiency, and is often referred to as the overkill effect.

On a local basis, with LET defined as being the energy deposited over a 1 μm section of track, this distance is appropriate for chromosomal radiation effects since it is roughly the width of a single chromosome.

#### Relationship between *Z* and LET_U_

The position of the turnover point can be estimated for different *Z* values. It is apparent from publications quoted above (15–18) that LET_U_ increases with *Z*, but the effect appears to saturate (i.e., further increase in LET have diminishing returns as far as the LET_U_ value is concerned). The Betha–Bloch equation for estimating the rate of energy loss with distance (*x*) traversed (dE/dx), which represents LET, contains a *Z*^2^ term in the numerator usually reflecting the charge of a fully electron stripped ion or proton. Larger *Z* values will also be associated with larger mass numbers and greater momentum with larger event volumes due to more complex nuclear collisions and energetic γ-ray emissions. Beyond the necessary critical dimension (be this radial or linear as a surrogate), biological killing efficiency will not increase if the event size becomes too large and physically beyond the individual chromosome. So, a saturation effect is to be expected. The smallest values of *Z* = 1 for a proton effectively reduces dE/dx, but the proton LET_U_ is only 30.5 keV/μm, suggesting that lighter charged particles exert more localized effects (caused by short range low energy secondary electrons). In this respect, the proton is more efficient at causing an increment in RBE with LET [but proton LET values are quite small, e.g., a LET of only 1–8 keV/μm in typical clinical exposures (26, 27) when using scanned proton beams may cause RBEs as high as 1.8 or more (8)].

The application of a simple differential equation can represent this process. Let us assume that *Z* is a continuous variable and if the initial rate of change in LET_U_ with *Z* is *S* and that this value then decreases in proportion to LET_U_ itself, representing a saturation effect controlled by the constant *k*, so that

(1) $$\frac{d\text{LE}{\text{T}}_{\text{U}}}{\mathrm{dZ}}=S-k\cdot \text{LE}{\text{T}}_{\text{U}}$$

which by integration of both sides and rearrangement leads to

(2) $$\text{LE}{\text{T}}_{\text{U}}=S∕k(\text{1}-\text{Exp}\left[\text{ -}k\text{(}Z\text{)}\right],$$

where *S/k* represents the maximum possible value of LET_U_.

Equation 2 can be normalized to the proton (*Z* = 1) LET_U_ of 30.5 keV/μm found by Belli et al. (16), so that for any *Z* a term *Z* − 1 is used such that:

(3) $$\text{LE}{\text{T}}_{\text{U}}=\text{3}0.\text{5}+S∕k\left(1-\text{Exp}\left[-k\left(Z-1\right)\right]\right)$$

This equation is used for data fitting purposes.

#### Changes in Radiosensitivities with LET

By increasing LET gradually, from the control low LET value of say clinical 4–6 MV photons (X-rays), we obtain small increases in the probability of additional LCBs; the energy deposition becomes maximally efficient (let this be represented by 100% efficiency for normalization purposes), and at higher LET values beyond LET_U_, the efficiency is reduced below 100% because of excess local energy deposition.

The separate relationship between α_L_ (the low LET control α value) and α_U_ (the value of α at the turnover point where LET = LET_U_) also exhibits saturation effects. In other words, the increment in α with LET show diminishing returns, since the lowermost α_L_ values have the highest gain in α. This effect is found with fast neutrons and with charged particle data, as shown in the Section “Results” below.

For an initial slope of *A* and a rate constant *j*, the rate of change of α_U_ with α_L_ will fall in proportion to α_U_, so that

(4) $$\frac{d\alpha_{\text{U}}}{d\alpha_{\text{L}}}=A-j\cdot \alpha_{\text{U}},$$

which leads after integration to:

(5) $$\alpha_{\text{U}}=A\text{/}j(1-\text{Exp }[-j\alpha_{\text{L}}]).$$

The β parameter can either be modeled in a similar way but with smaller overall changes, or to simplify matters for tentative modeling purposes, it could be assumed to be invariant at low doses where β-related cell kill is small. The data of Weyrather et al. (17) show that β values rises from a control value of 0.026 Gy^−2^ to a maximum of 0.044 Gy^−2^ in V-79 cells, and likewise from 0.02 to 0.42 Gy^−2^ for the CHO cells (α/β value of 0.192 Gy^−2^ in one instance must be artifactual due to the fitting program), i.e., by up to a factor of around two, which is small compared to the maximum increments in α with LET of around 10. There is more abundant data for 64 MV fast neutrons where β undoubtedly increases (22). Although such neutron experiments will probably underestimate the maximum possible rise in α and β, since the neutron LET spectrum (and its average value) may not necessarily be close to the LET_U_ for an ionic beam. Nevertheless, further analysis of these data, which compare neutrons with megavoltage X-rays show fits of β_neu_ = 1.54 β*_x_* _−_ *_ray_* or β_neu_ 0.097 [1 − Exp (23.6 β*_x_* _−_ *_ray_*), as will be shown below]. The experimental variation in such data is considerable and the two fitted equations were obtained after elimination of: repair deficient cells (where α > 0.6 Gy^−1^), or where neutron β values close to zero, or if the increment in β exceeded that in α (suggesting experimental artifact). It should be noted that α_U_ and β_U_ values will be higher than the maximum values obtained for fast (64 MV) neutrons, and so the neutron data cannot be used directly to determine RBE changes for ion beam data.

A similar “saturation” function, is used to link β_L_ with β_U_, as given elsewhere (8):

(6) $$\beta_{\text{U}}=\left(R∕u\right)\cdot \left(\text{1}-\text{Exp}\left[-u\beta_{\text{L}}\right]\right).$$

Where *R* = 2.5 and *u* = 25, which provides a modest increase in *b*, and is compatible with the limited data discussed already, and with a maximum ceiling value of 0.1 Gy^−2^ for β_U_.

#### Obtaining α_H_ and β_H_ values

In simple mathematical terms, a discontinuous or biphasic (efficiency followed by inefficiency) model can be used, where for LET values up to that of LET_U_, increasing efficiency is represented as a linear simple proportional relationship, as used by Wilkens and Oelfke for protons (6), and where the α value at any LET higher than the control and lower than the turnover value will be

(7) $$\alpha_{\text{H}}=\alpha_{\text{L}}+\frac{\text{LE}{\text{T}}_{\text{x}}-\text{LE}{\text{T}}_{\text{C}}}{\text{LE}{\text{T}}_{\text{U}}-\text{LE}{\text{T}}_{\text{C}}}\cdot \left(\alpha_{\text{U}}-\alpha_{\text{L}}\right)$$

where α_H_ is the α value at any particular LET value (LET_x_) between the control and ultimate value of LET_x_ [which represents any LET value between the control value of LET_C_ (where α is α_L_)] and LET_U_, where the maximum α of α_U_ occurs.

For the initial linear portion of the relationship, there will be a uniform gradient of

(8) $$\frac{\alpha_{\text{U}}-\alpha_{\text{L}}}{\text{LE}{\text{T}}_{\text{U}}-\text{LE}{\text{T}}_{\text{C}}}$$

between the value of LET_C_ and LET_U_, which fulfills the requirement for linearity in this range of LET. It follows that, for example, if LET_C_ and LET_U_ are 1.2 and 120 KeV/μm respectively, with α_L_ and α_U_ of say 0.3 and 1.3 Gy^−1^, then for a LET_x_ value of 60, the process is only (1.3–0.3)/(120–1.2) × (60–1.2), which is close to being 50% efficient, and for a LET_x_ of 90, the efficiency will be (1.3–0.3)/(120–1.2) × (90–1.2), which is close to 75% efficiency.

In this way, the efficiency of cell kill per unit dose will increase linearly with LET, leading up to maximum efficiency (defined as 100%) at LET_U_.

For values of LET beyond the turnover point (where LET > LET_U_), the additional energy transferred does not contribute to extra lethality, but is wasted. That is, the excess energy (LET_x_ − LET_U_) beyond the optimal released energy is wasted. Consequently, inefficiency, expressed in energy terms by (LET_x_ − LET_U_)/LET_U_ increases. To express this in terms of efficiency, the relationship of: % efficiency = 100 − % inefficiency is used, and the α_H_ value is then scaled between α_U_ and α_C_.

Accordingly, the equation for α_H_ for LET > LET_U_ then changes to be:

(9) $$\alpha_{\text{H}}=\alpha_{\text{L}}+\left(1-\frac{\text{LE}{\text{T}}_{\text{x}}-\text{LE}{\text{T}}_{\text{U}}}{\text{LE}{\text{T}}_{\text{U}}}\right)\cdot \left(\alpha_{\text{U}}-\alpha_{\text{L}}\right)$$

which effectively expresses the reduction in α with increasing LET. In this way, if LET_x_ is 180 and LET_U_ is 120, the value of α_U_ at the turnover point of 100% efficiency will fall to 1 − (180 − 120)/180, which provides around 67% efficiency. For a LET_x_ of 240, we obtain 1 − (240 − 120)/240, which is 50% efficient. These efficiencies are of course relative to a normalized value of 100% at the turnover point.

Similar equations are used to provide β_H_, by proportionate scaling between β_L_ and β_U_. These are obtained by simply replacement of α_L_, α_H_, and α_U_ by β_L_, β_H_, and β_U_ respectively in Eqs (7) and (9).

#### Reduction of RBE with Dose

The reduction in RBE with reduced surviving fraction and increasing dose is obtained by the solution of the following iso-effect equation for high and low LET radiations at a dose *d*_L_ and *d*_H_, for low and high LET respectively:

(10) $$\alpha_{\text{L}}d_{\text{L}}+\beta_{\text{L}}{d_{\text{L}}}^{2}=\alpha_{\text{H}}d_{\text{H}}+\beta_{\text{H}}{d_{\text{H}}}^{2}$$

The solution for *d*_L_ is then divided *d*_H_ to provide the RBE, as shown in other publications (8, 24, 25).

For clinical iso-effect calculations, the solution of the following biological effective dose (BED) equations are used for the low and high LET:

(11) $$n\;d_{\text{L}}\left(1+\frac{d_{\text{L}}}{{\left(\frac{\alpha }{\beta }\right)}_{\text{L}}}\right)=m\;d_{\text{H}}\left(\text{RB}{\text{E}}_{\text{Max}}+\frac{{\text{RBE}}_{\text{Min}}^{2}\cdot d_{\text{H}}}{{\left(\frac{\alpha }{\beta }\right)}_{\text{L}}}\right),$$

where *n* and *m* are the respective numbers of fractions for the low and high LET.

The RBE parameters are replaced by LET (and the new parameters given in the sequence of equations described above) and then solved for *d*_H_. Total doses to provide the same BED can then be calculated for different numbers of fractions.

Computer programs using Mathematica (Champagne, IL, USA) software were constructed using the above equations.

## Results

**Figure 1:**
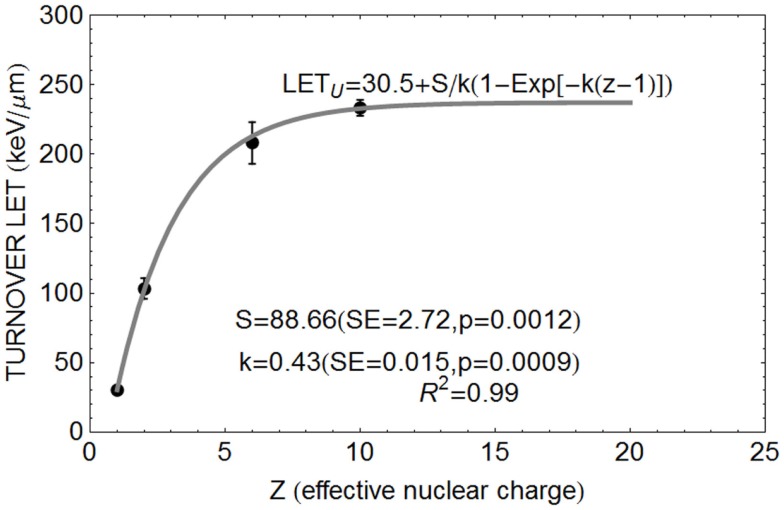
**Data points for relationship between *Z* and turnover point LET value LET_U_ with fitted parameter values based on Eq. (3)**.

The relationship between *Z* and LET_U_ shown in Figure 1, using pooled data for proton, helium, carbon, and neon ions (13–16), were fitted by Eq. (3).

**Figure 2:**
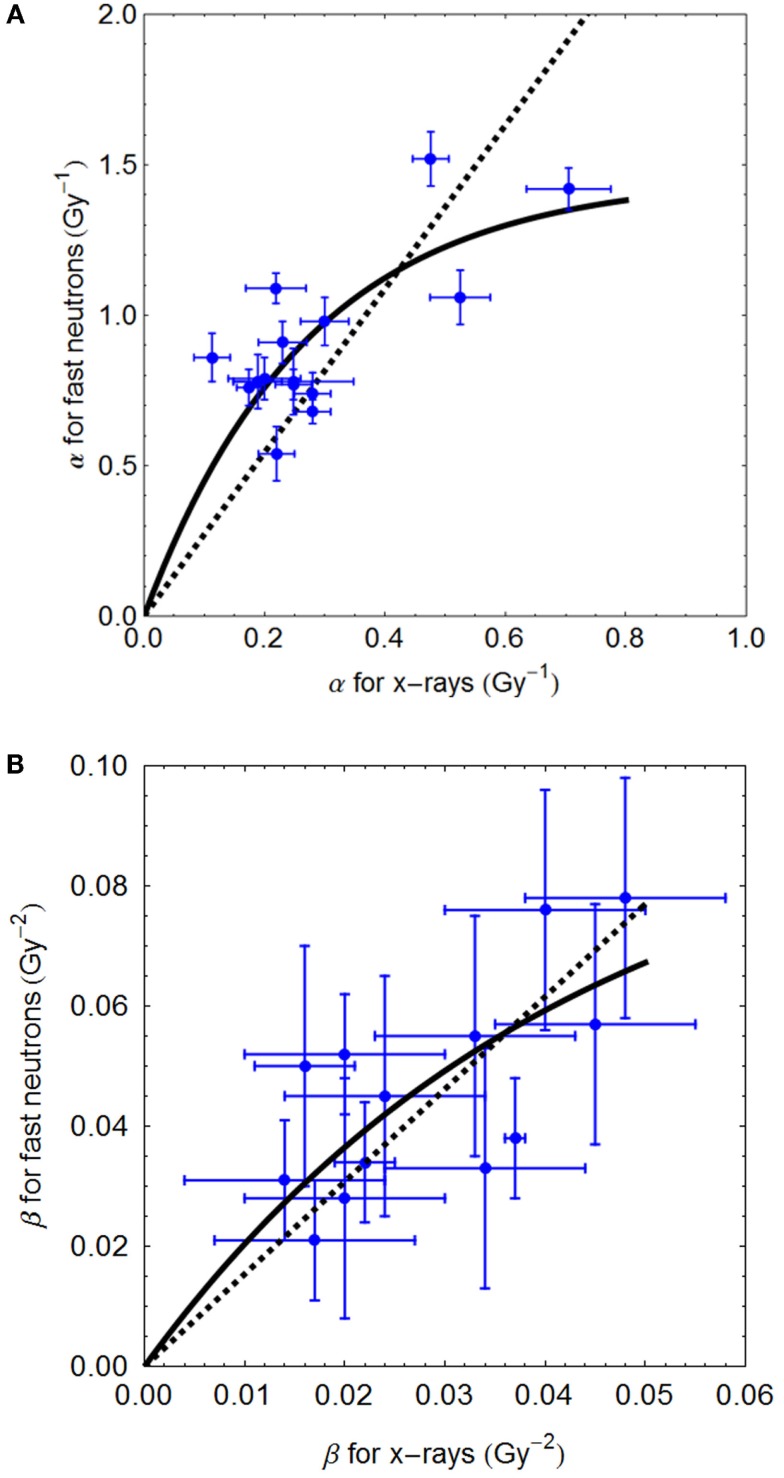
**(A,B)** Sixty-four megavolt fast neutron relationships between low and high LET radiosensitivity parameters. Linear no-intercept and non-linear least squares fits are respectively: **(A)** α_H_ = 2.72α_L_ and α_H_ = 5.37/3.68 (1 − e^−3.68αL^); **(B)** β_H_ = 1.57⋅β_L_ and 2.29/23.57(1 − e^−23.57 βL^) using Mathematica software.

The Clatterbridge fast neutron data (21), show the relationship between α_L_ (for values up to 0.8 Gy^−1^) and α_H_, and between β_L_ and β_H_, are shown in Figures 2A,B, respectively. In each case, the linear and non-linear fits are not significantly different (*p* > 0.05), although the residuals are smallest for the non-linear equations, which also have the advantage of not extrapolating to infinitely high radiosensitivity values.

**Figure 3:**
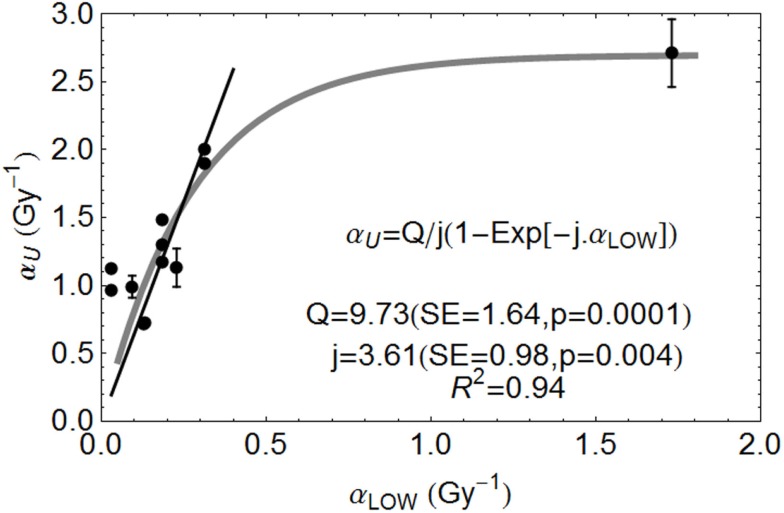
**Ion beam relationships between radiosensitivity parameters at low and high LET at the turnover point (***α***_H_ is here ***α***_U_) and fitted by the parameters shown, using Mathematica software**. Error bars are not available for all data used. Reproduced with permission from Ref. (8).

The relationship between α_L_ and α_U_ for various ions are shown in Figure 3, fitted to data from the literature [with data where negative β values obtained excluded]. The fitted equation is shown in the figure, but also with a least squares fit for a linear no-intercept relationship of α_U_ = 6.47 α_L_ (*p* < 0.001, *R*^2^ = 0.899) for α_L_ values less than 0.35 Gy^−1^, the more radio-resistant part of the radiosensitivity spectrum.

### Fits to experimental RBE data sets

**Figure 4:**
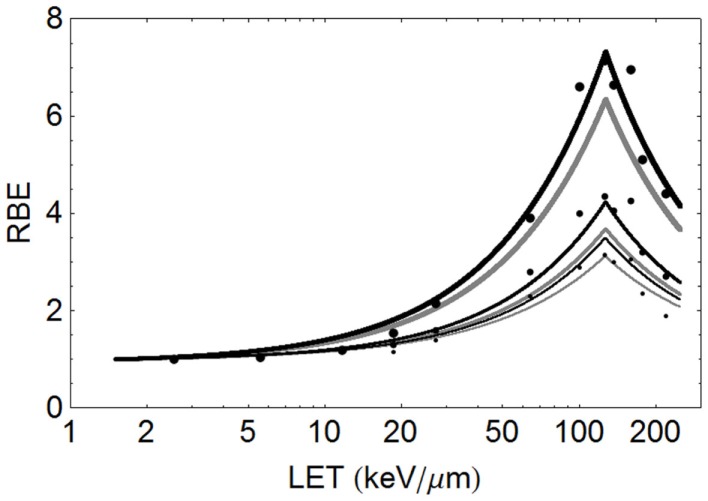
**Mono-energetic alpha particle data of Barendsen (with large points indicating 50% survival, medium sized points 10% survival, and the smallest points 5% survival, using the proposed model to provide fit lines, with black indicating use of parameters ***α***_L_ ***=*** 0.16 Gy^***−***1^, ***α***_U_ ***=*** 1.31 Gy^***−***1^, ***β***_L_ ***=*** 0.046 Gy^***−***2^, ***β***_U_ ***=*** 0.15 Gy^***−***2^ and gray using parameters ***α***_L_ ***=*** 0.15 Gy^***−***1^, ***α***_U_ ***=*** 1.35 Gy^***−***1^, ***β***_L_ ***=*** 0.03 Gy^***−***2^, ***β***_U_ ***=*** 0.08 Gy^***−***2^**. The thickest lines are for 50% survival, medium lines for 10% survival, and thinnest lines for 5% survival.

**Figure 5:**
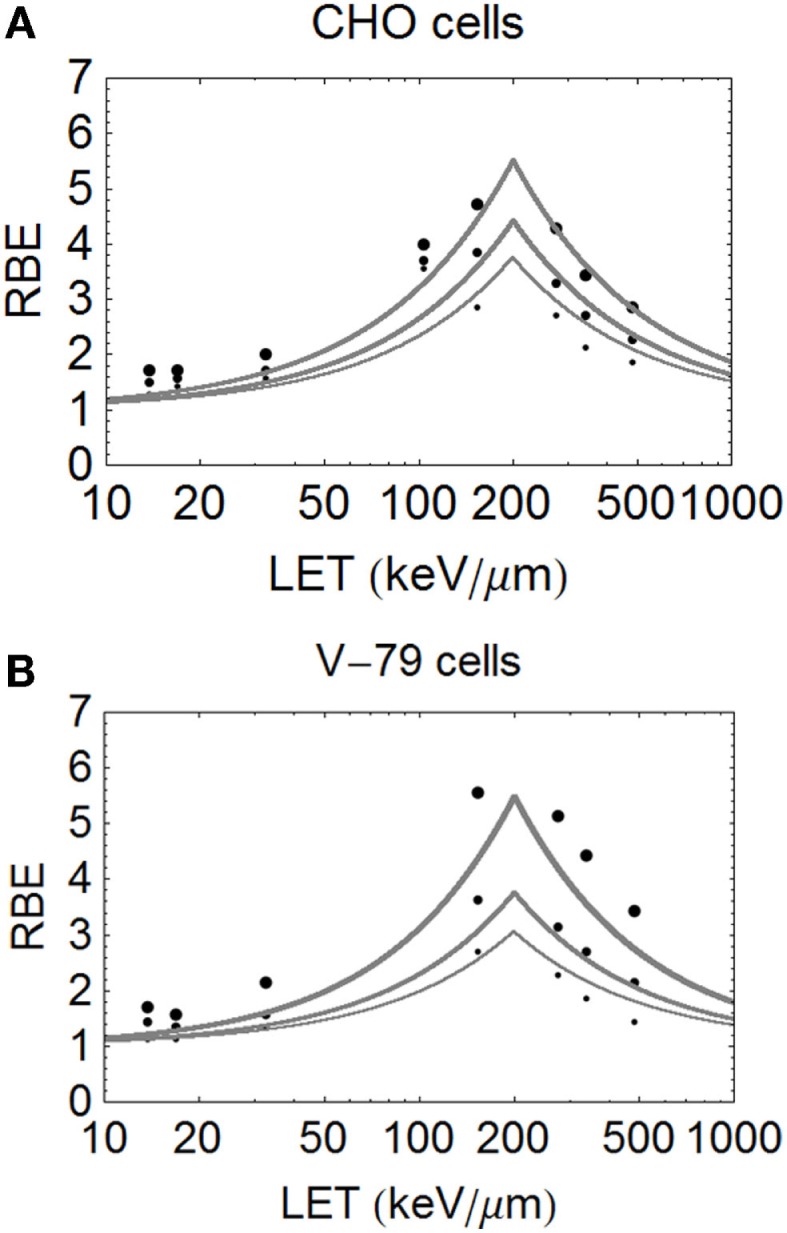
**(A,B)** Model fitted data of Weyrether et al. for C ions for three different cell lines and doses, coded in the same way as for Figure 4 with respect to line thickness and surviving fraction **(A)** for CHO cells and **(B)** for V-79 cells.

**Figure 6:**
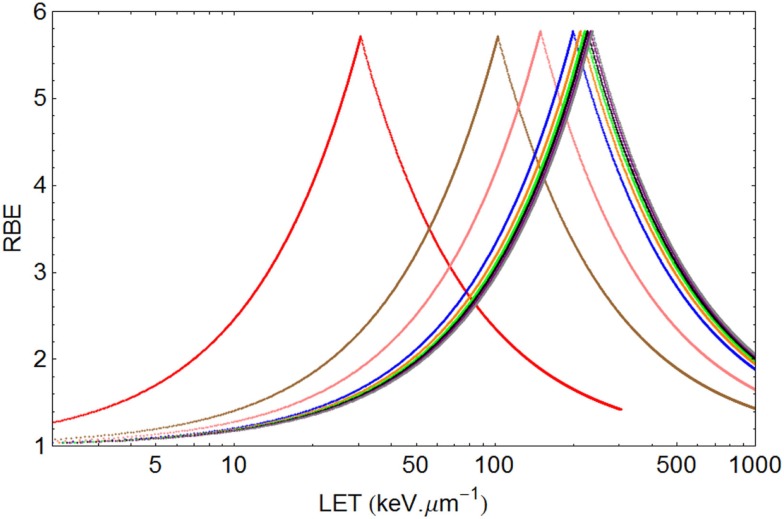
**Graphical displays of RBE and LET with unique turnover point positions for multi-ion data of Todd, assuming ***α*** ***=*** 0.14 Gy^***−***1^ and ***β=*** 0.05 Gy^***−***2^ for a dose of 1.5 Gy**. From left to right the ionic elements are shown as follows, with color code and LET_U_ (rounded to nearest integer for values over 100) in parentheses: Deuterium (red, 30.5), Helium (brown, 103), Lithium (pink, 150), Boron (blue, 200), Carbon (orange, 213), Nitrogen (green, 221), Oxygen (Black, 227), Neon (purple, 232), and Argon (gray, 237).

**Figure 7:**
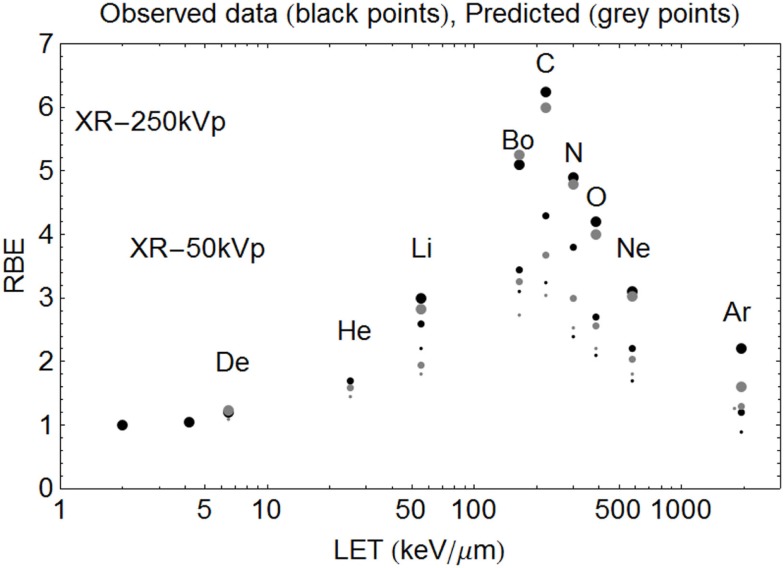
**Model predictions shown with data of Todd using the new *Z*-based model to determine LET_U_ and ***α***_U_, ***β***_U_ respectively, with LQ model correction for dose**. The largest sized points are for SF = 50%, the intermediate sized points are for SF = 10%, and the smallest sized points are for SF = 1%. The ions used and their LET (keV/μm) values are given respectively in parentheses. Deuterium (6.5), Helium (25), Lithium (55), Boron (165), Carbon (220), Nitrogen (300), Oxygen (385), Neon (580), and Argon (1940). Observed RBE data are printed as black points, with estimated RBE values as gray points. Starting on the left hand side the first two black points are for 250 and 50 kV X-rays respectively, followed by deuterons etc.

The model is superimposed to the experimental data sets, using different cell lines, of Barendsen (Figure 4) and Weyrather et al. (Figures 5A,B) and Todd (Figures 6 and 7).

The data of Barendsen used mono-energetic deuterium or helium (alpha) particles in one human cell type, with highly symmetrical curves which turnover at around 110 keV/μm. In this case (see Figure 4), the model fits the data reasonably well at all levels of surviving fraction. However, since this data set exists as plotted graphical surviving fraction results without access to the original data, there is inevitable uncertainty in assessing the low and high LET α and β values, which make the RBE determination even more difficult. The plot was obtained by assessment using α_L_ = 0.16, α_U_ = 1.31, β_L_ = 0.046, β_U_ = 0.15 obtained by crude measurements of survival curves and RBE plots, each on a logarithmic and linear scales, drawn by artists and which contain displacements of many data points for convenience of display, but the data set is better fitted by α_L_ = 0.15, α_U_ = 1.35, β_L_ = 0.03, β_U_ = 0.08, as shown in Figure 4. The Barendsen data set suffers from retrospective inaccuracies in estimating parameters from diagrams in publications rather than use of the raw data, but the graphic shows the sensitivity of the model to the input parameters.

The critical dependency of each RBE limit on the ratio of α and β at low and high LET respectively, demonstrates the importance of obtaining the most accurate possible data, rather than depending on published material which does not contain precise surviving fraction outcomes. The Barendsen data set also suggests a higher value of LET_U_ for alpha particles than obtained above using the formula based on *Z* in pooled data, at around 127 instead of 103 keV/μm; also the α_U_ is predicted to be 1.18 Gy^−1^ by Eq. (3). This illustrates the uniqueness of each data set and the distorting effect of pooling of data from different laboratories using different cell systems etc.

The important carbon ion data of Weyrather et al. (17), from GSI, which covers a broader range of LET values, shows an apparently constant turnover point for different cell types and surviving fractions (Figures 5A,B). The data are published with the LQ radiosensitivities, although the ions have a small variation in their LET spectrum (with a maximum spread of less than 5% for the highest LET values which reduces further with decreasing LET). So, it is unlikely that energy and LET spread contribute to the deviations from the modeled curves seen at lower LET values. The RBE values found at low LET values seem higher than expected, possibly due to biological sample variation, especially since irradiations were performed using two different accelerator systems (for LET values above and below 100 keV/μm) in different laboratories and presumably at different times. These data, although very informative, inevitably contain greater heterogeneity than the data of Barendsen, and the data are less well fitted. Another more stochastic approach is to use a Poisson function, which will be presented in a further publication.

In the case of Todd’s multi-ion data (24), a range of different mono-energetic ions were used (protons, deuterium, helium, lithium, boron, carbon, nitrogen, oxygen, neon, and argon), which implies that there will be at least nine different curves, one for each *Z* value, and each with unique turnover points. Such heterogeneous data were fitted surprisingly well by allocating a unique turnover point for each ion species, before estimation of the RBE, as shown in Figure 6, followed by the RBE estimations for each ionic species in Figure 7.

### Clinical radiobiology

**Figure 8:**
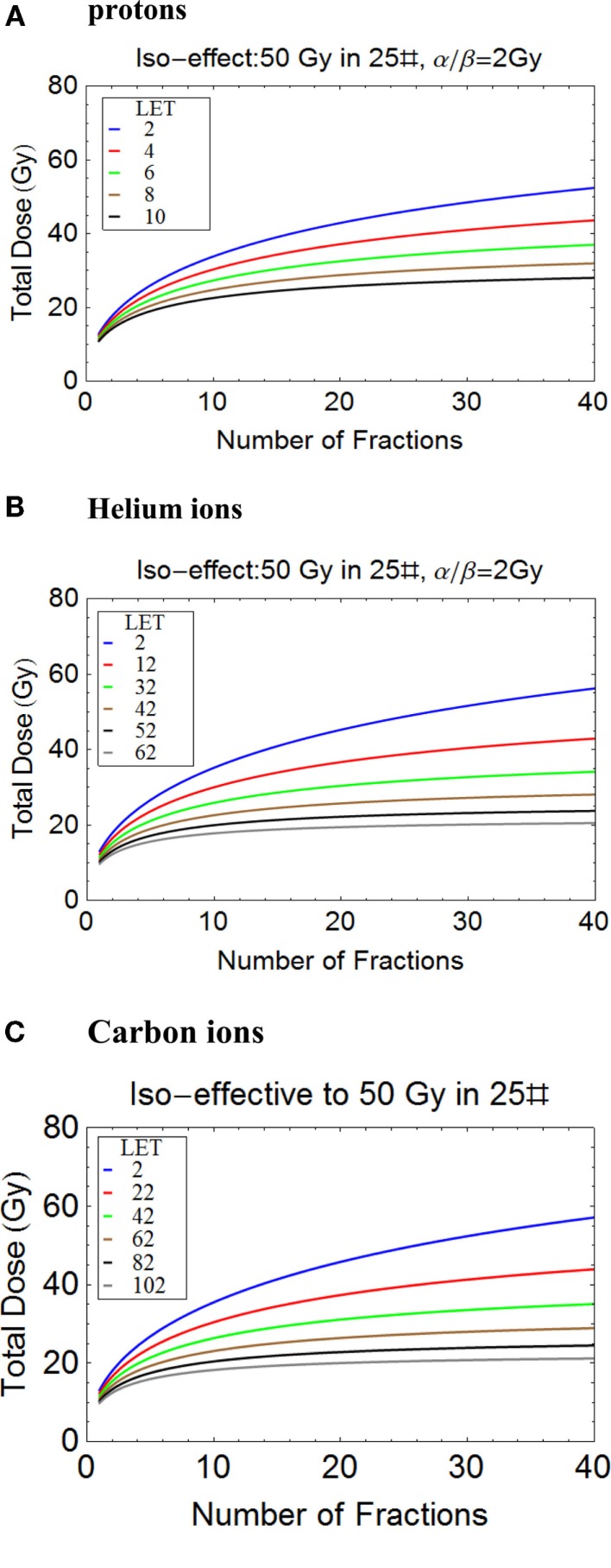
**(A-C)** Plots of total iso-effective dose versus number of fractions for the given iso-effect and α/β ratio. **(A)** Protons, **(B)** Helium ions, **(C)** Carbon ions.

It is possible to tentatively assess changes in total dose required for different fractionation schedules using protons, helium, and carbon ions, as shown in Figures 8A–C. The variations in LET are representative of the wide expected clinical ranges for non-Bragg peak regions and spread out Bragg peaks of different sizes and for scanned beams. It should be noted that the changes in total dose required with number of fractions (and consequently dose per fraction) are remarkably similar for the respective LET ranges used. This indicates the importance of LET mapping as well as dose mapping in the clinic, since RBEs and consequently changes in total dose with fractionation can be the same for a wide range of ions, as determined by their *Z* value and LET.

## Discussion

Simple differential equations which model saturation effects are commonly used in the physical sciences and in biology, with notable examples in pharmacokinetics. Saturation in the radiation context applies to the relationship between the effective event size and the bio-target. Maximum efficiency represents the maximum cell killing effect caused by locally absorbed energy, which differs from the energy released, some of which may be wasted by causing more local damage than is necessary to cause lethality, or is dissipated over a wider than necessary critical volume.

The new model offers a relatively simpler semi-empirical mathematical method for assessing changes in RBE with LET than has previously been available and provides a second order approximation. It can be more easily understood and used by clinicians, biologists, and others, without recourse to more complex mathematics. Also, the two saturation-based assumptions made, in comparison, are fewer than the assumptions required in other RBE models. For highly controlled and relatively homogenous data sets, this deterministic approach provides reasonable estimates of RBE. For protons, a variant of this approach has been published recently (8). The model depends on the assumptions of the LQ model where α and β are high level parameters, being ultimate coefficients of radiation induced cell death, rather than basic components of radiation effect such as DNA strand breaks etc.

The model is not intended to supplant existing models of RBE, but to be complementary. It would be highly advantageous in clinical practice if more than one model could be used, with clinical decisions allowed to proceed if at least two are in reasonable agreement. Thus, the LEM, MKM, and variants of the Katz models should continue to be used, and compared with the new model.

Improved input data would undoubtedly further improve the accuracy of the model. Rather than attempt to fit historical data, which are limited in terms of accurate determination of “maximum efficiency” turnover points, it would be better to conduct rigorous experiments to test hypotheses connected with the above models, such as the relationship of the initial slope to more precise estimates of the turnover point position (LET_U_) in different ions. This also requires a further stochastic interpretation necessary to match a range of LET values as would be encountered in many clinical beams. There is ample scope for research in this respect.

Some authors have emphasized the inverse association between low LET α/β and the final RBE (7, 13). This follows since α/β reflects repair capacity and intrinsic radiosensitivities, and is valid more at low doses. From the definitions of RBE_max_ and RBE_min_, it is easy to show that the former will be inversely related to related to (α/β)_L_, but the latter directly proportional to the square root of (α/β)_L_ (21, 28). The former assumption can be used for low dose per fraction treatments, where RBE_max_ dominates the RBE. The need to include changes in β with LET is necessary for estimations of RBE at higher doses, and where α/β is small as in human late tissue effects. The new model also preserves the overall symmetry of the curves at increasing dose. Accurate estimation of β from cell survival curves, especially when α values are large, are notoriously difficult to achieve. Our knowledge of how β changes with increasing LET is less well documented than for the larger and easier to measure changes in α with increasing LET. Only by meticulously conducted large scaled experiments, with greater than usual numbers of cell survival experiments, can these parameters be estimated to greater and sufficient accuracy.

Since neutrons are uncharged, they do not fall easily into this model, although the main products of neutron interactions such as recoil protons and other ions do, such that a spectrum of LET values will result, which in principle could be translated into RBE using the modeling described in this report. Again this would require further specific study.

There is considerable scope for the application of simpler RBE predictive models. Ideally prospective experiments should be performed with specific attention to LET–RBE turnover point position for different ions, the initial slope of the increment in RBE and the maximum value of α and β relative to their low LET values. These need to be determined for extensive *in vitro* libraries of human cell lines and, if confirmed, extended to more complex *in vivo* experiments. A single international center would be ideal for this purpose, as has already been proposed at CERN (29, 30). There, it might be possible to create a new extensive data base for LET–RBE relationships, and to re-confirm or refute the basic RBE principles listed on p. 3. Of special concern are the slopes of the relationship, and improved accuracy for key LET_U_ parameter, using multiple ion species in an appropriate panel of human cell lines, and to a much higher degree of accuracy than previously obtained. In this way, the data shown in Figures 1 and 3 could be enhanced by experiments on multiple ion species. Also, the results of all available models should be compared in such a single laboratory.

Such a project must be regarded as “essential science” for informing clinical practice, so that the best outcomes from particle therapy may in the future be fully, rather than partially, realized. Many practical enigmas remain within particle therapy (8, 31).

It is noteworthy for medical scientists to realize that in the first 6 weeks of the experiments that lead to the discovery of the theoretically predicted Higgs Boson, the entire laws of particle physics were not only re-confirmed, but to a much higher level of accuracy than previously achieved. Similar goals must be attempted in radiobiology, although over a longer time frame. This would provide the tools for greater predictive accuracy to particle radiotherapy, to improve its efficacy, as well as provide enhanced knowledge for human radiation protection, including astrobiology.

## Conflict of Interest Statement

The author declares that the research was conducted in the absence of any commercial or financial relationships that could be construed as a potential conflict of interest.
