# Corrigendum: A Simpler Energy Transfer Efficiency Model to Predict Relative Biological Effect for Protons and Heavier Ions

**DOI:** 10.3389/fonc.2016.00032

**Published:** 2016-02-18

**Authors:** Bleddyn Jones

**Affiliations:** ^1^Department of Oncology, Gray Institute for Radiation Oncology and Biology, Oxford, UK

**Keywords:** RBE, protons, ions, radiotherapy, radiobiology

An error was caused by inaccurate transcription of one equation from the computer programmes in the above paper (1). On page 4 of the above article, in the paragraph before Eq. 9, the biological ‘inefficiency’ should be expressed by (LET_x_ − LET_U_)/(LET_x_ − LET_C_), that is the local energy deposition (LET_x_) in excess of the maximum efficiency energy deposition (LET_U_), divided by the local energy deposition that exceeds that imparted by the control radiation (LET_C_).

This means that Eq. 9 should be modified to be:

$$\alpha_{\text{H}}=\alpha_{\text{L}}+\left(1-\frac{{\text{LET}}_{\text{x}}-{\text{LET}}_{\text{U}}}{{\text{LET}}_{\text{x}}-{\text{LET}}_{\text{C}}}\right)\cdot (\alpha_{\text{U}}-\alpha_{\text{L}})$$

The author apologises for this error, although is pleased to state that the graphical displays were all achieved using the correct equation as given above.

## Conflict of Interest Statement

The author declares that the research was conducted in the absence of any commercial or financial relationships that could be construed as a potential conflict of interest.
